# Antileishmanial activity of a formulation of 2-n-propylquinoline by oral route in mice model

**DOI:** 10.1051/parasite/2011184333

**Published:** 2011-11-15

**Authors:** N. Campos Vieira, J. Vacus, A. Fournet, R. Baudouin, C. Bories, B. Séon-Méniel, B. Figadère, P.M. Loiseau

**Affiliations:** 1 Groupe Chimiothérapie Antiparasitaire, UMR 8076 CNRS, Faculté de Pharmacie, Université Paris-Sud Rue Jean-Baptiste 92296 Châtenay-Malabry Cedex France; 2 Société Drugabilis 5, rue Jean-Baptiste Clément 92290 Châtenay-Malabry France; 3 IRD US 084, Laboratoire de Chimie des Substances Naturelles, Faculté de Pharmacie rue Jean-Baptiste Clément 92296 Châtenay-Malabry cedex France

**Keywords:** leishmaniosis, quinoline, antileishmanial, drug formulation, leishmaniose, quinoléine, substance antileishmanienne, formulation

## Abstract

2-n-propylquinoline is presently a drug-candidate for the treatment of visceral leishmaniosis in pre-clinical development. As this compound is in an oily state, it needs to be formulated and the objectives of this study are: to prepare a formulation; to demonstrate that the new salted formulation did not alter the activity of the active ingredient; and finally, that this activity was quite good compared to the reference oral drug, miltefosine. Therefore, a 2-n-propylquinoline formulation, as camphorsulfonic salt, was prepared and characterised. On the *Leishmania donovani* / Balb/c mice model, a treatment by oral route at 60 μmoles/kg/day for ten consecutive days with this formulation was compared to 2-n-propylquinoline alone and to miltefosine, the oral reference drug. The salt formulation did not alter the activity of the 2-n-propylquinoline. The formulation reduced the parasite burden of 76% compared to 89% for miltefosine (not significant). The characteristics of this formulation results in a suitable drugability of 2-n-propylquinoline for further studies.

## Abstract (fr)

La 2-n-propylquinoléine est un candidat-médicament pour le traitement de la leishmaniose viscérale en développement préclinique. Ce composé se trouvant à l’état huileux, une formulation adéquate est nécessaire, et les objectifs de cette étude sont de mettre au point cette formulation, de démontrer qu’elle n’altère pas l’activité du principe actif et que cette activité est suffisamment bonne par rapport à celle du médicament de référence utilisé par voie orale, la miltéfosine. Une formulation de 2-n-propylquinoléine sous forme de sel d’acide camphosulfonique a été sélectionnée et caractérisée. Après administration de cette formulation par voie orale à la dose de 60 μmoles/kg/jour pendant dix jours consécutifs sur le modèle de leishmaniose expérimentale à *Leishmania donovani* chez la souris Balb/c, par comparaison avec la miltéfosine, les réductions de la charge parasitaire observée ont été respectivement de 76 % et 89 %. Les caractéristiques de cette formulation de la 2-n-propylquinoléine permettent de l’envisager pour des études ultérieures.

Leishmanioses are tropical and sub-tropical parasitic diseases affecting more than 12 million people in the world and for which the chemotherapy is limited by toxicity of the drugs such as antimonials, and by the emergence of drug resistance (WHO, 2007). Despite significative advances during this last decade with the use of AmBisome®, a lipid formulation of amphotericin B, and miltefosine, the first orally active drug, resistance is at risk since it has been obtained in laboratory by selecting drug-resistant parasites under *in vitro* drug pressure (Mbongo *et al.*, 1998; Seifert *et al.*, 2003). It is therefore necessary to find new chemical classes having antileishmanial activities. Thus, the 2-substituted quinoline series, isolated from *Galipea longiflora* (Rutaceae), a Bolivian tree used for the treatment of cutaneous leishmaniosis lesions by the native Chimane Indians, was intensively studied (Fournet *et al.*, 1993). More than 130 compounds have been synthesized and evaluated *in vitro* and *in vivo* against various *Leishmania* species. Some of them were active on experimental leishmaniasis models by oral route (Nakayama *et al.*, 2005; Fournet *et al.*, 1996). The synthesis of these compounds has a low cost and their *in vivo* activity on experimental leishmaniasis models prompted “Drug for Neglected Diseases Initiative” to enter this series in its preclinical development pipeline.

In a previous study, we compared three compounds in regard of their easiness of synthesis, their chemical stability, as well as their *in vivo* antileishmanial activity and toxicity, and we proposed 2-n-propylquinoline (Fig. 1), the natural compound, as the most promising for further investigations (Campos-Vieira *et al.*, 2008). However, the oily state of the native free base cannot allow the development of a simple solid dosage form such as tablet. The compound needs therefore to be formulated.

**Fig. 1.**
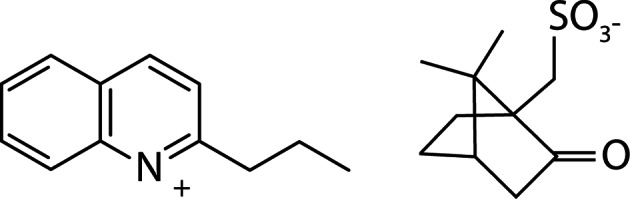
Chemical structure of the 2-n-propylquinoline camphorsulfonic salt.

The objectives of this study are: to prepare a formulation, to demonstrate that the new salted formulation did not alter the activity of the active principle, and finally, that this activity was quite good compared to the reference oral drug, miltefosine. Therefore, we report on the set up of a crystalline salt and on its *in vivo* antileishmanial activity on a *Leishmania donovani* / Balb/c mice model after oral administration.

## Material and Methods

### Chemicals

2-n-propylquinoline was synthesized by previously described procedures (Fakhfakh *et al.*, 2003). Physical and spectral data including proton and carbon-13 nuclear magnetic resonance and mass spectrometry were used to check the purity of 2-n-propylquinoline. Miltefosine (hexadecylphosphocholine or HePC) was provided by Zentaris laboratories (Frankfurt, Germany).

### Selection and Preparation of 2-N-Propylquinoline Formulation

#### Screening and selection of the suitable salt

Six acids were tested for their capacity to form a crystalline salt when associated to 2-n-propylquinoline: benzensulfonic, camphor-sulfonic, methanesulfonic, sulfuric, nitric, toluenesulfonic. These acids were selected on the basis of their pK^a^. Various crystallization media including ethanol, isopropyl alcohol, acetonitrile and water were used to obtain crystals from equimolar mixtures of the drug and the acids.

#### Physico-chemical characterisation

At the end of the crystallization step, the resulting solids were analyzed by optical microscopy and powder X-ray diffraction. For optical microscopy analysis, small samples of the solids isolated after crystallization in the different media were observed by a Navitar 12× Zoom microscope or a Leica DMIRB inversed microscope (Nanterre, France), both equipped with a digital camera and a motorized stage. Microscopy images were recorded either under direct light or between crossed polarizer and analyzer. X-ray powder diffraction (XRPD) analysis was performed on a Brüker-AXS D8 Advance diffractometer (Brücker, Paris, France), using a copper anti-cathode, a mono-crystalline silicon sample holder and a position sensitive detector.

### *In Vivo* Antileishmanial Activity

The formulation was evaluated *in vivo* for its antileishmanial properties by oral route on the *Leishmania donovani* / Balb/c mice model, comparatively to 2-n-propylquinoline alone and miltefosine, the oral reference drug, according to previously described protocols (Nakayama *et al.*, 2005; Nakayama *et al.*, 2007). Six- to eight-week-old Balb/c mice (Élevages Janvier, Le Genest Saint Isle, France) were infected intravenously on day 1 with 107 *L. donovani* (MHOM/ ET/67/HU3) amastigotes derived from spleen hamsters and randomly sorted into three groups of ten and one group of 12. The treatment started one week postinfection, on day 8, and continued for ten consecutive days untill day 17. One group of ten mice received orally 100 μl of the formulation, dissolved in 1 % carboxymethylcellulosis, the second group of ten mice received 100 μl of a suspension of 2-n-propylquinoline in 1 % carboxymethylcellulosis, and the third group of ten mice received 100 μl of miltefosine, dissolved in 1 % carboxymethylcellulosis. Each group was treated orally and daily at 60 μmoles/kg of body weight. The fourth group of 12 mice was treated with 100 μl of 1 % carboxymethylcellulosis as a control. At day 24, all groups were sacrificed and livers and spleens were weighed. Parasite numbers were determined by counting the number of amastigotes/500 liver cells in Giemsa-stained impression smears prepared from the liver and multiplying that value by the weight of the liver in milligrams (Nakayama *et al.*, 2005). The mean number of parasites per gram of liver of treatment groups and controls were compared using Student’s t test or the Kruskal-Wallis nonparametric analysis of variance test for comparing two groups. Significance was established for a P value < 0.05.

## Results and Discussion

**Fig. 2.**
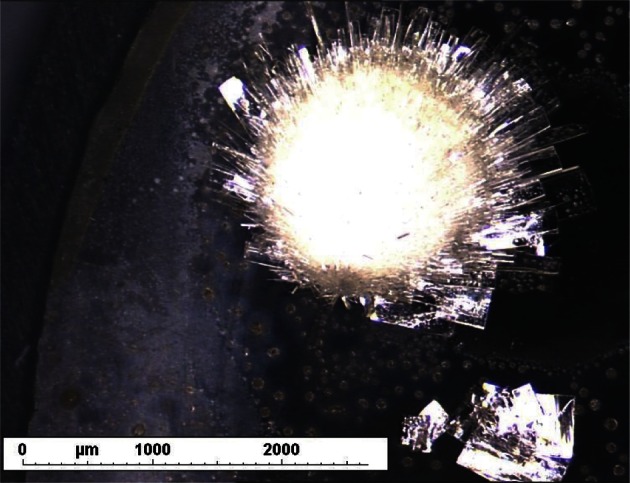
Optical microscopy photography of the 2-n-propylquinoline formulation (× 40).

**Fig. 3.**
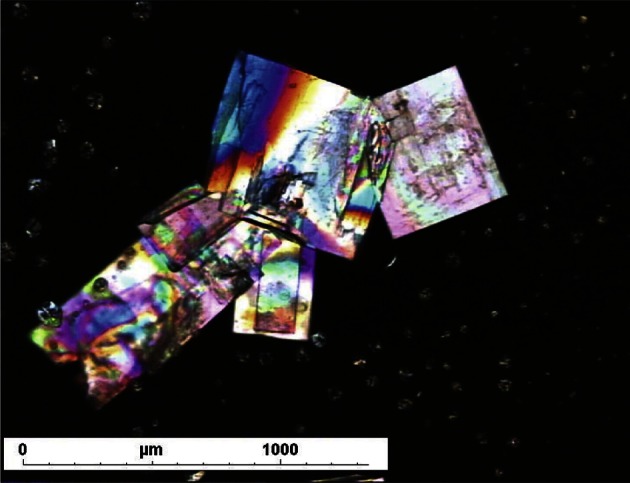
Polarized light optical microscopy photography of the 2-npropylquinoline formulation (× 81).

**Fig. 4.**
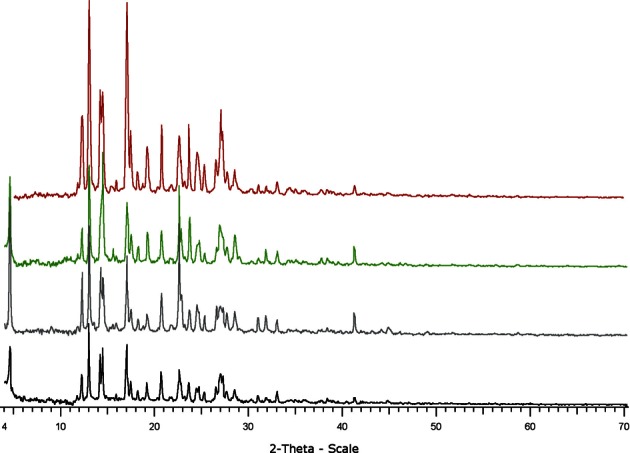
– X-ray diffraction patterns of four batches of the 2-npropylquinoline camphorsulfonic salt.

The chemical structure of the 2-n-propylquinoline camphorsulfonic salt is reported on Fig. 1. From the various attempts to obtain a crystalline salt with the tested acids, only the camphorsulfonic sample was shown to contain regularly shaped and birefringent particles (Figs 2 and 3). During the whole study, different batches of the camphorsulfonic salt were produced. Their crystalline forms were compared by means of powder X-ray diffraction. They all presented the same diffraction pattern (Fig. 4), showing that they were made of the same crystal form.

**Fig. 5.**
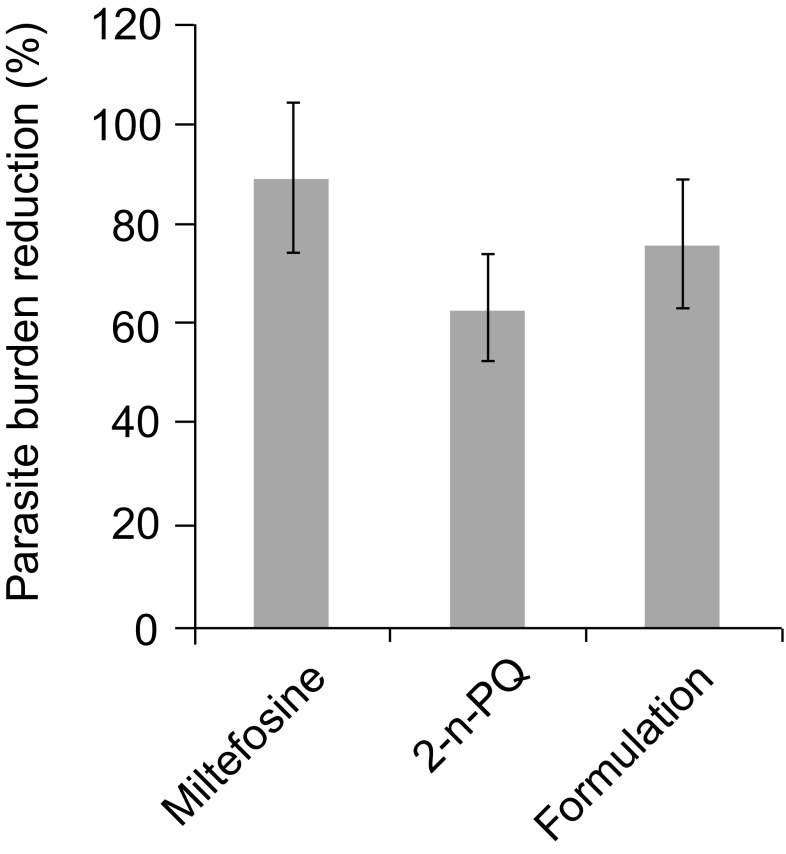
*In vivo* antileishmanial activity of the 2-n-propylquinoline camphorsulfonic salt *vs* 2-n-propylquinoline (2-n-PQ) *vs* miltefosine on the *L. donovani* / Balb/c mice model. - Miltefosine *vs* 2-n-PQ: no significant (P > 0.05). - 2-n-PQ *vs* formulation: no significant (P > 0.05). - Miltefosine *vs* formulation: no significant (P > 0.05).

The presence of the camphorsulfonic salt did not significantly modify the antileishmanial activities of 2- n-propylquinoline (Fig. 5). Camphorsulfonic acid alone did not exhibit any *in vitro* antileishmanial activity on the *L. donovani* intramacrophage amastigote model at 150 μM (data not shown).

After a treatment with the salt formulation by oral route at 60 μmoles/kg/day for ten consecutive days corresponding to 10.3 mg of 2-n-propylquinoline/ kg/day, the parasite burden was reduced in the liver by 76 % whereas the parasite burden reduction after treament with miltefosine at the same dose and in identical conditions was 89 % which was not significantly different (Fig. 5).

In summary, from the 2-substituted quinoline series intensively studied for its antileishmanial activity since about many years, 2-n-propylquinoline, the natural compound, is a suitable candidate for further investigations (Campos-Vieira *et al.*, 2008). However, the major limitation for further investigations was the oily state of the native free base that would have prevented the development of a simple solid dosage form. Identifying a solid form such as crystalline camphorsulfonic salt of the compound and having proved that the selected material did not decrease the compound efficacy *in vivo* now allows coming back to that option. From a first pharmacokinetics study described by Iglarz *et al.*, 1998, the present formulation makes now possible the determination of pharmacokinetics parameters in optimized conditions. Moreover, these data could help to define treatment regimens in experimental leishmaniosis models by associating 2-propylquinoline, that exhibits a short half-life, with miltefosine, having a long half-life, in order to prevent drug resistance to both the compounds.
